# Current Challenges in Pediatric Asthma

**DOI:** 10.3390/children11060632

**Published:** 2024-05-24

**Authors:** Andrija Miculinić, Iva Mrkić Kobal, Tin Kušan, Mirjana Turkalj, Davor Plavec

**Affiliations:** 1 Children’s Hospital Srebrnjak, Srebrnjak 100, 10000 Zagreb, Croatiamturkalj@bolnica-srebrnjak.hr (M.T.); 2 Clinic for Pediatric Medicine Helena, Ulica Kneza Branimira 71, 10000 Zagreb, Croatia; iva.mrkic@gmail.com; 3 Faculty of Medicine, Josip Juraj Strossmayer University of Osijek, Josipa Huttlera 4, 31000 Osijek, Croatia; 4 Faculty of Medicine, Catholic University of Croatia, 10000 Zagreb, Croatia; 5 Prima Nova, Healthcare Institution, Zagrebačka Cesta 132A, 10000 Zagreb, Croatia

**Keywords:** asthma, children, phenotypes, diagnosis, therapy, follow-up

## Abstract

Asthma is a chronic lung disease characterized by reversible bronchoconstriction and inflammation of the bronchi. Its increasing prevalence in childhood as well as different triggers make asthma a challenging disease in several ways: defining its phenotype/endotype, the diagnostic approach (especially in younger children), therapeutic options, and systematic follow-up. Considering these problems, this review approaches the current status and limitations of guidelines used for asthma management in children. It also emphasizes the key points which could lead to a better understanding and the direction to take in future studies.

## 1. Introduction

Asthma is defined as a chronic lung disease presenting with a variety of symptoms that arise from the same pathophysiological mechanism: the narrowing of the airways due to contraction of smooth muscles of the bronchi (bronchoconstriction) and the edema and secretion of the mucosal membrane. The resulting spasm and resistance of the airways causes air to become trapped distally from the obstruction and does not allow for adequate ventilation of alveolar spaces, leading to increased breathing workload.

Although the main mechanisms of asthma are well known there is constant debate regarding how endotype and environmental factors modify possible triggers and disease severity [1]. There is also still no consensus about the diagnostic and therapeutic approach for all different phenotypes/endotypes. Difficulty in performing standardized lung function testing in younger children poses a considerable challenge when assessing recurrent wheezing episodes or chronic cough, especially in patients younger than the age of five years [2].

Both the definition and the available medications are debatable. There are many possible treatment options, but of the newer ones, only a few have been tested in children and are therefore still unavailable for everyday use in smaller children [3]. Finally, after the diagnosis has been made, a follow-up plan for each patient on a regular basis must be followed because the disease is variable and needs adjustment of therapy and lifestyle changes.

A comprehensive search was conducted using the Web of Science and PubMed databases, specifically targeting publications in the English language within the last five years using the following keywords: paediatric asthma, challenges, difficulties, diagnostic, therapy, follow-up.

## 2. Asthma Phenotypes/Endotypes

Although asthma was previously considered a single disease, today, we know of several phenotypes with different triggers, which would more accurately define it as a syndrome [4]. It also seems that phenotypes/endotypes do not consistently predict disease prognosis and reflect only temporal variability in airway inflammation [5]. We also know that not all children follow the same pathway of asthma/allergic disease development and that an important feature for multimorbidity is single or multiple allergen sensitization.

Today, the most common clinical phenotype in children (and adults) is allergic asthma [6]. Here, the main mechanism is eosinophilic inflammation provoked by allergens—usually inhaled particles like pollen or dust mite feces. After the contact with the mucosal membrane of the airways, an IgE-mediated activation of eosinophils causes their degranulation. The whole mechanism is much more complex, and many proinflammatory cytokines are involved in the process [7]. There is still no definite proof for the main cause of the global increase in allergic diseases, but researchers and clinicians both consider lifestyle and environmental changes to be the most plausible explanation for this phenomenon [8].

Other than allergic triggers, other known causes include viral infections, exercise, some medications (acetylsalicylic acid), cigarette smoke, and changes in humidity. Asthma provoked by these triggers is also called nonallergic or intrinsic asthma, which is then further subdivided into more specific groups based on their main characteristics, primarily their triggers, molecular mechanisms (endotypes), and therapeutic response [9]. Their onset is much more likely to occur during adulthood, with a predominance in female patients and the tendency of exacerbations having a higher degree of severity [10].

Regarding the diversity of phenotypes/endotypes, it is progressively becoming more difficult to classify these groups and subgroups of asthma. To date, there is still no definite classification. The global initiative for asthma (GINA) classified asthma mainly on the level of symptoms, airflow limitation, and lung function variability. However, experts have acknowledged that this division into four categories based on the level of symptoms alone is no longer viable for making decisions regarding treatment options, but it may still have its uses when patients are initially assessed [11,12,13].

Another significant layer involves individual features reflected by differences in genetic transcription and inflammatory molecular responses. These can be either caused by mutations or epigenetic factors (methylation of DNA segments) that cause different expressions of genes. There are several candidate genes known to be connected to increased risk of asthma development. Researchers mainly focus on protein-coding genes, even though there is a clear relevance of non-coding RNA in the pathogenesis of asthma [14,15]. Genetic research is promising, but we know that asthma is not solely a genetic disease and is instead a unique expression of the interactions between genetic and environmental factors [16]. Although a genetic classification would be most appropriate and precise, it would be very unpractical for everyday use for clinicians.

In the last few years, researchers have invested significant effort into defining as many triggers as possible of asthma-type predictors rather than genetic mechanisms. This is because the latter may be more confusing than useful in everyday clinical practice (as explained earlier) [17]. Still, there is no evidence that the triggers themselves are enough to predict childhood asthma, as they often overlap or even change, especially during the transition to adulthood [18].

Some research papers have addressed the problem of allergen exposure and the development of allergic asthma and showed the connection between early life exposure and atopy [19,20,21]. This matches the theory that the time and type of exposure play a key role in the Th2 immunologic response. Inheritance by parent of origin did not seem to impact the development or severity of the disease [22]. It seems that some interactions of endotoxins with TLR-4 pathways are associated with a higher risk of asthma development [23]. However, some studies have shown a difference in risk for developing asthma according to genotype, as there are also “protective” genes that are not affected by exposure at all [24]. Another interesting prospective study investigating the development of asthma and atopy in high-risk infants proved that environmental factors play an important role in predisposed children. However, allergen avoidance during pregnancy and early infancy is inconclusive. The authors suggest a multifaceted approach with the inclusion of different diagnostic tests such as IgE levels and skin prick testing as predictors [25].

There are some types of asthma that do not respond well to standard therapy [26]. These types can be classified as severe or difficult-to-treat asthma. It is noteworthy that difficult-to-treat asthma does not necessarily mean that the asthma is severe, but that other factors like therapy adherence and comorbidities have an impact on the suboptimal control of the disease. A very serious form of severe asthma is called Brittle asthma. It was first defined in 1977 and describes a type of asthma with very variable peak expiratory flow (PEFR) despite high doses of inhaled corticosteroids [27]. This form has two distinct types: Type I is characterized by persistent daily chaotic variability in PEFR (greater than 40% variation more than 50% of the time). Type II has sudden, unpredictable, and potentially life-threatening falls in PEFR with otherwise well-controlled symptoms. Newer literature disregards this nomenclature, possibly because it could be easily defined as another asthma phenotype [28].

There are still debates about other clinical features like viral-induced wheezing, which may or may not be asthma. Due to the fact that many children with viral-induced wheezing will come out as completely healthy individuals over time, it is obvious that making predictions in early infancy could lead to overdiagnosis and possibly unnecessary treatment [29].

## 3. Diagnostic Approaches

**Figure 1:**
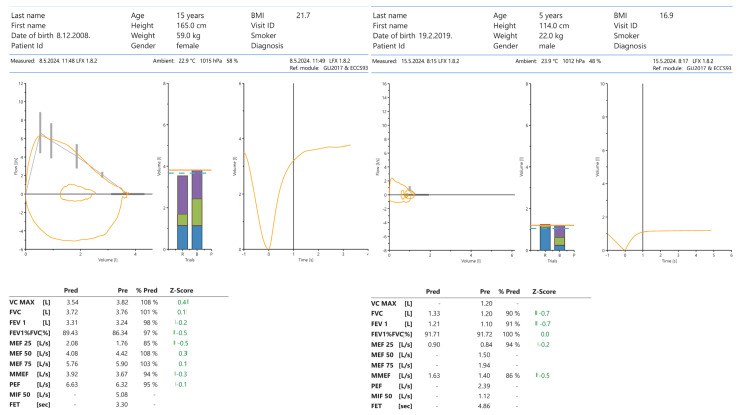
Differences in spirometry values in children of different ages.

The challenge with the diagnostic evaluation of children with asthma is not a new one. The current guidelines rely mostly on lung function testing as the standard for detecting reversible bronchoconstriction. However, this is not possible for all age groups, as the reproducibility of the test relies on patient cooperation. Smaller children, especially those under the age of five years, are commonly confused by the instructions given by technicians and other medical personnel. They may also become anxious and unwilling to perform the given task [30]. As such, expected values of forced expiratory volume in the first second of expiration (FEV1) and forced vital capacity (FVC) cannot be attained and give us the wrong impression that the patient has severe obstructive/restrictive respiratory impairment compared to values in adolescents and adults (Figure 1). There is also a much greater variability in the results because of differences in chest wall compliance and somatic growth [31]. In adolescence, there is increased variability of normal values due to greater differences between biological and chronological age and exchanging phases of grow and development.

Some medical institutions have the means to partly overcome this obstacle by applying other methods of lung function testing such as high-oscillation techniques (i.e., impulse oscillometry—IOS) which do not measure respiratory volumes or flow but the resistance of the airways. With IOS, there is no need for active cooperation of the patient, but unfortunately, there are no clear validated cutoff values for children [32]. Only lists for predicted values exist to date [33]. 

There are still promising efforts regarding the use novel diagnostic methods, including Ventica^®^, light plethysmography, and WheezeScan^®^ by Omron Healthcare, Kyoto, Japan [34,35,36]. These try to overcome the limitations of standardized tests but are not available to all clinical and research centers. Still, they are a welcome addition to other tests and should be taken into account if no other means to make a definitive diagnosis are at hand. In the future, if adequate reference values can be calculated, these novel methods might even become the new standard for asthma diagnostics in small children.

In addition to lung function testing, there are also other methods used for asthma diagnosis and follow up. One of these is measuring inflammation of the lungs. Nitric oxide is known to be a molecule released from tissues when inflammation is present and can therefore be detected in exhaled air in patients with eosinophilic bronchial inflammation, as is the case in patients with asthma [36]. Measuring the fraction of exhaled NO (FeNO) is thus a parameter that is useful in monitoring children with asthma (again, only in cases when they cooperate during test performance). Still, FeNO might be elevated in several conditions that are also a part of the multimorbidity of asthma: atopic dermatitis, food allergies, etc., and the extent of elevation does not reflect disease severity [37,38]. FeNO can be low in some conditions, although inflammation might be present, i.e., exposure to tobacco smoke [39,40]. Therefore, NO fraction is not a good enough biomarker to be taken as definite proof of asthma, and it also cannot be used rule out the possibility of its presence [41]. Direct methods for measuring inflammatory reactions like bronchoalveolar lavage, biopsy, and induced sputum are more precise because they correlate much better with inflammation, especially regarding the decline in eosinophils when treatment is successful. They are rarely used because of invasiveness and limited compliance in smaller children [42]. Clinicians must choose the most beneficial and least invasive method, so in cases when it is possible, FeNO measurement should be considered [43].

Another important feature of asthma is hypersensitivity of the lungs. This can be measured using strictly standardized bronchoprovocation testing; for example, with methacholine. There are several reasons why this type of lung function test is important. Firstly, airway hyperresponsiveness may be the only objective proof of airway dysfunction [44]. Secondly, the degree of hyperresponsiveness corelates well with disease severity [45]. And finally, hyperresponsiveness in individuals with asthma is a good predictor of clinical course to sort out patients who really need attention and regular treatment (even when clinical remission is present) [46].

Although bronchoprovocation tests are highly recommended, performing these examinations requires cooperation of the examinee. It is also not without risks because bronchoconstriction is deliberately provoked. If standardized protocols are followed, the risk can be minimized [47,48]. Still, the age at which the testing is performed poses a considerable restriction [47].

In low-income countries, diagnostic tests are much less likely to be performed due to limited availability, and symptom-based diagnosis is likely to result in misdiagnosis—either inclusion of healthy/non-asthmatic children or failure to detect those with less typical symptoms. There is also the need for better-equipped and available medical centers that provide professional support and adequate treatment options [49]. Although the limited funds may seem like the weakest link, the main problem is not only the lack of financial resources but also the small number of low-income countries that have a national asthma plan for children [50]. In situations where no other option is available, it is suggested to perform at least peak expiratory flow measurements, while reference values may be calculated easily using a formula that correlates well with PEF in emergency settings [51].

## 4. Therapeutic Approaches

The center of interest of all diseases is the possibility of treating them. Because asthma is a chronic disease, treatment options tend to be directed towards longer use and a wider age range. Medications target two somewhat different phases of asthma: maintaining control and relieving symptoms when they arise. Firstly, there is the so-called prophylactic treatment. This means that patients are given medications that need to be taken regularly as a means to avoid worsening of the disease. These medications are called controllers. In case of worsening of symptoms, or so-called asthma exacerbations, there are other medications that try to lower asthma symptoms and treat potentially life-threatening situations. These are called relievers and are given mainly during a shorter period of time, only when symptoms or discomfort appear [52]. In some cases, if asthma symptoms regress or sometimes even completely disappear and no signs of chronic inflammation are present, we can consider the asthma to be in remission, a state of a disease when, theoretically, no medications are needed because no symptoms/inflammation of the airways are present [13].

As good control of symptoms in children is essential, GINA proposed the step-up system as the preferred way of adjusting therapy type and doses according to disease severity and symptom frequency. There are five steps with different main and alternate suggested therapy options, ranging from very low doses of only inhaled corticosteroids to biological therapy combined with medications from previous steps.

Asthma in children is highly variable and is driven by the underlying airway inflammation, which was not addressed until recently by the GINA Step 1 treatment. Previously short-term SABA use alone was supposed to be given periodically when dyspnea or cough appeared. GINA 2019 has introduced a significant and pertinent update in the management of asthma exacerbations. After considering numerous studies on the harmful effects of short-acting beta agonists (SABAs) alone, it has been determined that there is now ample evidence to recommend against using SABA alone in the treatment of children and adolescents with asthma. Those guidelines added low-dose inhaled corticosteroids as an add-on to SABA as the basis for treatment of children with asthma and anti-inflammatory relievers (AIR, fixed combination of formoterol with inhaled corticosteroid) for the treatment of adolescents. This has remained the same, even in the latest GINA guidelines [12,53,54].

When Step 2 care is required, regular daily low-dose ICS may be recommended, but still with no guidance on changes in treatment based on clinical status. Step 2 care also comes with a recommendation to add short-acting β2-agonists (SABA) as needed, seen by many patients as the ‘core’ asthma medication because of symptom relief [55]. Another option is the daily use of leukotriene receptor antagonists (LTRA).

Many parents/caretakers have safety concerns about regular use of an ICS, such as restricted growth in children. Short-term use of high doses of inhaled or systemic corticosteroids did not impact linear growth [56], and minimal changes were observed when regular low-dose inhaled corticosteroid therapy was used in children with persistent asthma [57,58]. There is also a study that showed that uncontrolled or poorly controlled asthma had a high negative impact on the general health conditions of children, as well as linear growth [59]. Still, this so-called “corticophobia” causes problems for clinicians due to non-adherence and avoidance of corticosteroid use.

Poor asthma control may be due to over-reliance on short-acting β2-agonists and underuse of inhaled corticosteroids (ICS), patterns often established early in the patient’s journey. Adolescents also tend to forget, or due to their innate tendency to confront authority deliberately, avoid the regular use of prescribed medications. One potential response to these challenges is the as-needed use of an ICS plus a fast-acting β2-agonist (i.e., SABA or fast-acting LABA) combination as an alternative to their usual as-needed SABA alone.

In GINA Step 3, inhaled corticosteroids are given in combination with long-acting β2-agonists (LABAs). Even though inhaled medications (ICS with or without LABA) are potent in reducing asthma symptoms and preventing exacerbations, the impact on the natural history of the disease and disease progression is relatively small [60]. Another medication that showed the potential to reduce asthma exacerbations is tiotropium. Tiotropium is a long-acting antagonist of muscarinic receptors (LAMA). It was first used to treat adults with chronic obstructive lung disease (COPD) and also showed a use in chronic lung inflammation. In a recent study, although tiotropium did not reduce mean daytime symptoms, it reduced exacerbation risk significantly compared with a placebo in children aged 1 to 5 years who previously had persistent asthma symptoms [61]. The long-term impact on disease control is also debatable, but it shows some degree of improvement regarding the prevention of airway remodeling [62]. Still, there is a lack of in vivo human clinical trials to prove this possibility, as to date, studies have only been performed on animal models and human smooth muscle tissue.

GINA Step 5 is considered only if all other asthma treatment options did not provide adequate asthma control. Therefore, many medications have been considered as possible candidates for this step of asthma treatment.

Oral corticosteroids are generally avoided as asthma controllers, as they block endogenous glucocorticoid production and show a high rate of systemic side-effects. Children on long-term oral corticosteroid therapy (such as those with juvenile idiopathic arthritis) also show signs of slower growth in height and may also develop insulin resistance and even iatrogenic Cushing’s syndrome in more severe cases [63,64]. Oral or parenteral glucocorticoids may still be used in phases of acute exacerbations (in combination with standard doses of relievers—β2-agonists and ipratropium bromide), and they have been found to lower patient hospital admission [12,65].

Biological therapy is still considered as one of the last lines of therapy. However, currently, we know that in certain phenotypes, this might be the sole way of reducing asthma symptoms. Therefore, biological therapy should be included earlier, especially if typical symptom and diagnostic features indicate that such patients would benefit from it [3].

Currently, biologic therapy is mostly focused on lowering the activation of effector cells [66]. Because the pathophysiology of allergic asthma is the most well-defined one, most of the biologic agents were developed as a means to block Th2-type inflammation of the bronchi. The mechanism of action of biologics is theoretically well defined, but in practice, it might surprise clinicians because of the high variability in patient response to treatment. For example, some patients were reported to develop antibodies to biologic agents, which caused a decline in medication potency [67]. Raising the dose in such cases could lead to severe adverse effects. This means that the benefits of this type of medication, although targeting specific known pathways of eosinophilic inflammation, have to be carefully adjusted and monitored for each patient individually. Here, we would like to mention the most notable types of biologic agents.

Omalizumab or anti-IgE antibodies are considered for the management of severe, atopic asthma in childhood. They have a proven benefit and only minor side-effects compared to other medications and are therefore largely approved as a good treatment option [68]. They have also been shown to be useful in treating other atopic diseases, mainly atopic dermatitis and as a premedication while performing oral immunotherapy. Randomized controlled trials are still being performed [69,70].

Dupilumab or anti-IL4R antibodies are the second most used biologic therapy for children with asthma. The medication proved to be safe and significantly improved disease control when other prescribed medications were regularly taken [71,72]. It proved to be even more efficient in treating sever atopic dermatitis [73].

Anti-IL5 is still being tested and has shown a good effect on disease control in children and adults when adjunct to standard care in people with severe eosinophilic asthma and poor symptom control [74].

Although all these medications proved useful, there is a need for better treatment stratification to guide which children might benefit from these treatments [68]. Because of this, many researchers suggest that the basis for prescribing biologic drugs should be established after careful consideration of endotypes and not only asthma severity [75]. The use of better biomarkers should also be addressed for therapy effect evaluation purposes. Also, biological therapy for asthma treatment is still not registered for children under the age of six years.

The GINA guidelines for treating childhood asthma may be regarded as a good first step in treatment but should be individually applied because of changes in asthma presentation. Much of the data for Steps 4 and higher are extrapolated from studies on adult patients and adolescents, but very few from children, especially preschoolers [76]. There are ongoing clinical trials addressing this issue.

## 5. Follow-Up

Even though most phenotypes of asthma tend to come into a state of remission, exacerbations cannot be foreseen. This is especially the case for asthma in childhood, as changes are more common. One such period when changes occur most frequently arises during puberty and adolescence. Research has shown that hormone activity changes the transcription of genes and might be the cause for immunologic changes in these age groups [77]. Because of the unpredictability of disease severity and long-term outcomes, patients have to undergo regular check-ups, at least once yearly, or even several times a year if the disease is not well controlled.

As the symptoms of asthma are not always present, and in many cases are not present when visiting a pediatrician, it is important that parents are properly educated about the typical and less-typical symptoms of asthma to be able to recognize and administer adequate medications to their children. Patient involvement and consulting is the most important factor that contributes to good asthma control [78]. To aid parents and caregivers, there are several possibilities regarding objective measuring tools for home use. The most basic one is performing peak expiratory flow measurements using a simple device. The values are written down, and in consultation with a clinician, interpreted to provide standard normal values for that patient. When symptoms arise or are suspected, the same device is used to check whether there is a possible obstruction that needs acute treatment. This, however, has not been shown to have an additional benefit compared to adequate bronchodilator use [79]. Another novel option is the use of digital wheeze detectors (like Omron WheezeScan), as some pilot-studies have shown that based on symptoms only, parents detected bronchial obstruction in their children only in 3.1% of cases [80].

Even when remission is supposedly achieved, patients might still have some degree of ongoing inflammation and airway remodeling [81,82]. Even bronchial hyperresponsiveness might persist in some individuals [83]. That being the case, there is a growing interest in redefining asthma remission. Generally speaking, asthma remission is defined as the absence of signs and symptoms for a longer period of time (at least 12 months). This definition is very vague and is not informative when considering whether to continue treatment. There is still no way we can tell that asthma has been healed or “grown out of”, because the pathophysiological mechanisms may still be present or can at least be reactivated by a trigger. Even in patients with remission, there might be a relapse. According to this, asthma remission should be divided into at least two groups: clinical remission (no symptoms) and complete remission, which requires the normalization of the underlaying pathology in addition to the remission of symptoms [53].

Asthma changes during childhood growth, which impacts the phenotype and endotype, making predictions of the clinical course harder [84]. Children and parents may perceive the absence of symptoms as a cause to cease treatment on their own without expert counselling. The challenge in this case lies in the ability and effort of clinicians to educate patients and parents alike about the benefits of regular asthma treatment even when no symptoms are seen. They should at least be encouraged to do regular check-ups, and diagnostic tests. On the other hand, asthma questionnaires help physicians to define asthma phenotypes, and the number of exacerbations may direct them to better treatment options if all the collected information is accurate. Therefore, expert advice should be given to all patients, and education programs should be included in regular follow-ups with feedback from children and parents alike.

## 6. Discussion

Considering all the addressed challenges in asthma definition, diagnosis, treatment, and follow-up protocols, we propose to improve asthma guidelines by implementing the following suggestions:

- Classification

All facts considered, asthma has varying phenotypical features that are affected by genotype, trigger exposure, and comorbidities. Its development is still unclear, hard to predict, and difficult to classify. The best approach is to use a classification most suited for the type of result that is expected, whether it is for research or a clinician’s treatment purposes.

Making a classification that reflects the most efficient method of treatment would be most beneficial, but with added exceptions for specific subtypes that should be considered.

- Diagnosis

A broader variety of diagnostic tests should be included, and standardized values should be calculated so that all age groups could benefit from adequate tests. These diagnostic tools should also be adjusted in accordance with patient age and cooperation.

To more easily define cut-off values for diagnostic tests, more and larger cohort studies must be performed (like for the high oscillation techniques—IOS).

There is a need for better biomarkers and parameters used to screen children with symptoms associated with asthma to sort out those who can be diagnosed having as asthma and needing treatment. The search for sensitive and more specific diagnostic tests is still ongoing.

- Treatment

The stepwise treatment approach proposed by GINA is still the most accurate one and is good for typical asthma phenotypes/endotypes (especially allergic asthma). There is little risk of unnecessary treatment, but it is too slow for more severe cases of asthma, as achieving the best treatment option requires time and careful monitoring.

Treatment should be adjusted individually by always considering possible risks if the duration needed to achieve remission would be too long. Earlier treatment interventions with biologic medications should be provided for patients with atypical symptoms with severe eosinophilic airway inflammation. This way, serious adverse events due to low levels of asthma control could be avoided.

Another level of disease control is the treatment of comorbidities. If only the asthma is treated, disregarding other connected medical conditions, the effects could be less than satisfactory. Therefore, if difficulty treating treat asthma is suspected, algorithms for comorbidity detection should be used. This is especially the case for children where all pathologic medical conditions with overlapping symptoms should be treated alongside each other.

Coordination with national and international medical regulatory agencies allows for a broader spectrum of medications because there is a lack of therapeutic options registered for use in smaller children (especially under the age of 2 years). This leads clinicians to be unable to prescribe medications that would counter clinical manifestations and/or prevent exacerbations.

- Follow-up

Once diagnosed, the most important part for every clinician is regular check-ups with diagnostic tests and screening for partially or uncontrolled asthma. This means at least one visit to the specialist per year, and in case of difficult-to-treat asthma, even more frequently. The search for new or not-yet-defined triggers is also important to allow doctors to give advice on needed lifestyle changes and adequate treatment [85].

If therapy is not efficient, clinicians should search for possible comorbidities or misdiagnoses, (like exercise-induced laryngeal obstruction).

Some asthma phenotypes show a silent loss of lung function that becomes more obvious during long-term follow-up. An example is aspergillus allergies and also other types with discrete but persistent inflammation and airway remodeling.

Clinicians should use asthma control questionnaires more often and educate parents and children through working groups and online educational materials. E-diaries and mobile technology might be a very useful way to aid patients and parents to monitor asthma on a regular basis. Patient expectations and fears that could be used to define their treatment goals should be included.

The challenges, solutions, and aims are summarized in the Table 1.

## 7. Conclusions

Asthma is a complex disease with different endotypes in infancy, childhood, and adolescence. Improvements in the classification of asthma at an early age, finding new biomarkers for a specific endotype, the development of adequate methods of lung function measurement, and precise patient-centered treatment and follow up are some of the key issues in improving pediatric asthma care.

## Figures and Tables

**Table 1:** Challenges, possible solutions, future research needs.

Challenges in Pediatric Asthma	Possible Solutions	Future Research
Classification	- Classification that reflects the most efficient way to start efficient treatment (with exceptions for specific subtypes)	- Studies on endotypes of asthma in early childhood - Biomarkers of early childhood asthma
Diagnosis (especially in children <5 years of age)	- Development and introduction of new lung function tests into clinical practice	- Development of new lung function tests - Improving existing lung function tests - Defining cut off values for children <5 for IOS *, FeNO **, nasal NO *** - Comparison of different techniques of FeNO measurement - Research on new biomarkers of early childhood asthma
Treatment (especially in children <5 years of age)	- Treatment should be adjusted individually taking, comorbidities into account - Fixed combination of drugs should be available (registered) for children under the age of 5 - Earlier entrance of biologics into therapy	- Studies on safety and efficacy of ICS + LABA **** or ICS + formoterol in children <4 years - Studies of tiotropium efficacy in children 1–5 years of age - Studies on biologics safety and efficacy in children <6 y
Follow-up (especially in children <5 years of age and adolescents)	- Regular specialist checkups - Education of GPs, parents, and caregivers - Regular lung function and FeNO monitoring - Introduction of eDiaries, new devices (like wheezescan), and mobile technology into follow up	- Comparing asthma control via regular specialist checkups vs. asthma control via GP check-ups - Assess the use of mobile technology in pediatric asthma follow-up

* Impulse oscillometry. ** Fraction of exhaled nitric oxide. *** Nasal nitric oxide. **** Inhaled corticosteroid + long-acting beta agonist.
